# Adiponectin, Leptin and Visfatin in Hypoxia and its Effect for Weight Loss in Obesity

**DOI:** 10.3389/fendo.2018.00615

**Published:** 2018-10-18

**Authors:** Linda K. Rausch, Maximilian Hofer, Stephan Pramsohler, Susanne Kaser, Christoph Ebenbichler, Sven Haacke, Hannes Gatterer, Nikolaus C. Netzer

**Affiliations:** ^1^Hermann Buhl Institute for Hypoxia and Sleep Medicine Research, Bad Aibling, Germany; ^2^Department Sports Science, University Innsbruck, Tirol, Austria; ^3^Department of Internal Medicine 1, Medical University of Innsbruck, Tirol, Austria; ^4^CD Laboratory for Metabolic Crosstalk, Department of Internal Medicine 1, Medical University of Innsbruck, Tirol, Austria; ^5^RehaMED GmbH, Herxheim, Germany; ^6^Institute for Mountain Emergency Medicine, Eurac Research, Bozen, Italy; ^7^Division of Sports Medicine and Rehabilitation, Department of Medicine, University Ulm, Ulm, Germany

**Keywords:** hypoxia, obesity, weight loss, adiponectin, leptin, visfatin, exercise

## Abstract

**Rationale:** Hypoxia induces leptin gene expression in human adipocytes via hypoxia-inducible factors (HIF-α/β). Under ambient moderate hypoxia, leptin in adipocytes is elevated for at least 14 days. Leptin is supposedly involved in the reduced food intake, increased utilization of fatty acids for energy production and possible weight loss observed at high altitudes. Literature on adiponectin and visfatin in high altitude is inconsistent with reports of elevated levels and non-elevated levels. Exercise in hypoxia studies in obese subjects have shown a significant weight loss after up to 3 weeks, but it is unclear if this effect holds up for longer time periods. Therefore, we aimed to investigate 32 obese subjects completing 52 exercise and rest sessions within 8 months at either moderate or sham hypoxia and to analyze leptin, adiponectin, and visfatin mRNA-expression at different time points of exposure.

**Methods:** Abdominal subcutaneous fat biopsies were taken from 32 obese subjects before, after 3 months and after 8 months of intervention. Subjects were randomly divided into two groups and exercised at moderate intensity at two different study sites twice a week. The IG was exposed to normobaric hypoxia (FiO_2_: 14.0 ± 0.2%,) at exercise and at rest (FiO_2_: 12.0 ± 0.2%) and the CG to sham hypoxia. Quantitative real-time polymerase chain reaction (qPCR) was used in order to determine mRNA-levels of leptin, adiponectin, and visfatin.

**Results:** No differences in leptin levels after 3 and 8 months compared to baseline and between groups were found. There was no significant difference regarding adiponectin or visfatin at any time point compared to baseline in the hypoxia group, but an increase after 3 months was seen in the control group at normoxia compared to the hypoxia group (adiponectin: *p* = 0.029 and visfatin: *p* = 0.014).

**Conclusion:** In this first several months' duration randomized sham controlled hypoxia exercise and rest study with obese subjects, we found no time extended leptin mRNA-expression in subjects under hypoxia after 3 and 8 months compared to baseline levels. Moderate exercise in normoxia not in hypoxia leads to elevated adiponectin and visfatin levels after 3 months.

## Introduction

Obesity as a global public health issue has been connected to an increased risk for comorbidities such as type 2 diabetes or cardiovascular disease. Metabolic complications of obesity correlate with subcutaneous adipose tissue layers in the abdominal, gluteal and femoral depots, which store over 80% of total body fat (1). The size of fat stores can vary from 5 to 60% of total body weight. In order to achieve a reduction of body weight as well as an improvement of adiposity-related metabolic dysfunctions, exercise training has shown to be effective (2–4). One form of exercise training, which leads to increased weight loss in obese subjects, is exercise training under hypoxic exposure. Previous studies have demonstrated that obese subjects training in a hypoxic environment for several weeks loose significantly more weight compared to obese controls training in normoxia (5–9). Likewise, Netzer et al., examined obese subjects performing an 8 weeks' intervention of mild physical exercise in normobaric hypoxia, which caused a significantly greater weight loss than exercise in sham hypoxia (6). A similar target group training in hypoxia, also showed significantly reduced serum lipid levels, which are measured for cardiovascular risk prediction (5). However, these were short-term investigations with a maximum intervention duration of 2 months. For the first time, we investigated the long-term effect of hypoxia exposure in our previous study combined with moderate intensity exercise over an 8-month period in obese people (10). We found no added effect by hypoxia on body composition and metabolic risk factors in the long term. Surprisingly, after 3 months of exercise, no further weight loss was detected in both, control- and intervention group. According to recent literature, the additional effect of short term hypoxia is linked to increased hormone expression of leptin when being exposed to a hypoxic environment, despite the reduction of body weight (8). In Lippl et al., elevated levels of leptin were accompanied by higher basal metabolic rate and improvements in body composition. Elevated levels of leptin are triggered by hypoxia-induced factors (HIFs) and are thought to reduce food intake due to appetite reduction at high altitudes (11, 12). Additionally, increased leptin expression leads to an enhanced reduction of fat absorption and fat storage, as well as to an enhanced increase of fatty free acids utilization for energy production (13, 14). Short term investigation of circulating leptin hormone expression in obese and normal weight subjects at altitude are mostly consistent, reporting an increase of such, but also a stagnation when acclimatized to altitude (13, 15). When looking at its' gene expression, we know, that leptin gene expression in adipocytes is regulated by a variety of physiological and experimental stimuli (16). Its adipocyte-specific expression was elevated, when measured in extracted and cultivated human adipocytes under moderate hypoxia, which lasted for more than 14 days with a peak level at day 10 (7). In the long-term, the duration of elevated leptin gene expression levels under the influence of hypoxia remains unclear.

Another adipocyte-specific hormone expression, which is strongly and inversely associated to adipose tissue mass, is adiponectin. Conditions such as obesity lead to a reduction of adiponectin, whereas intense exercise seems to have no effect on adiponectin concentration in healthy normal weight persons (17, 18). Concerning changes of adiponectin during exercise interventions under hypoxia, studies only investigated healthy and non-obese subjects for a short time of stay. Likewise, Barnholt et al. (19) report no significant differences of adiponectin in a group of healthy non-obese persons sent to high altitude compared to a group remaining at sea-level for 3 weeks. Contrary to those findings, a study of a 9-day high altitude exposure to climbers found a significant increase of adiponectin. This might be related to stimulation of substrate oxidation for energy usage (20).

Concerning obesity treatment and the related containment of inflammatory processes, visfatin, an adipokine, is additionally to mention because it is found at elevated levels in obese people as a mediator of endothelial dysfunction and cardiovascular diseases (21). Literature on visfatin expression in human cells exposed to hypoxia is still very scarce. The accumulation of visfatin correlates with the expression of HIF-1α (22). In animal studies, the visfatin mRNA expression was found to be up-regulated when rat hepatic stellate cells were exposed to hypoxia for 12 h and the visfatin protein level was elevated after 6 h of hypoxia exposure (23, 24).

To the best of our knowledge, this is the first long-term interventional training study with obese subjects under the exposure of hypoxia investigating mRNA alterations of visfatin, adiponectin and leptin levels of abdominal subcutaneous fat cells. Based on illustrated literature and our previously published results of the same cohort (10), we hypothesized that in the long term, there is no change in leptin mRNA expression, but elevated levels of adiponectin- and visfatin mRNA expression when resting and exercising in hypoxia rather than in normoxia.

## Methods

### Participants

**Figure 1:**
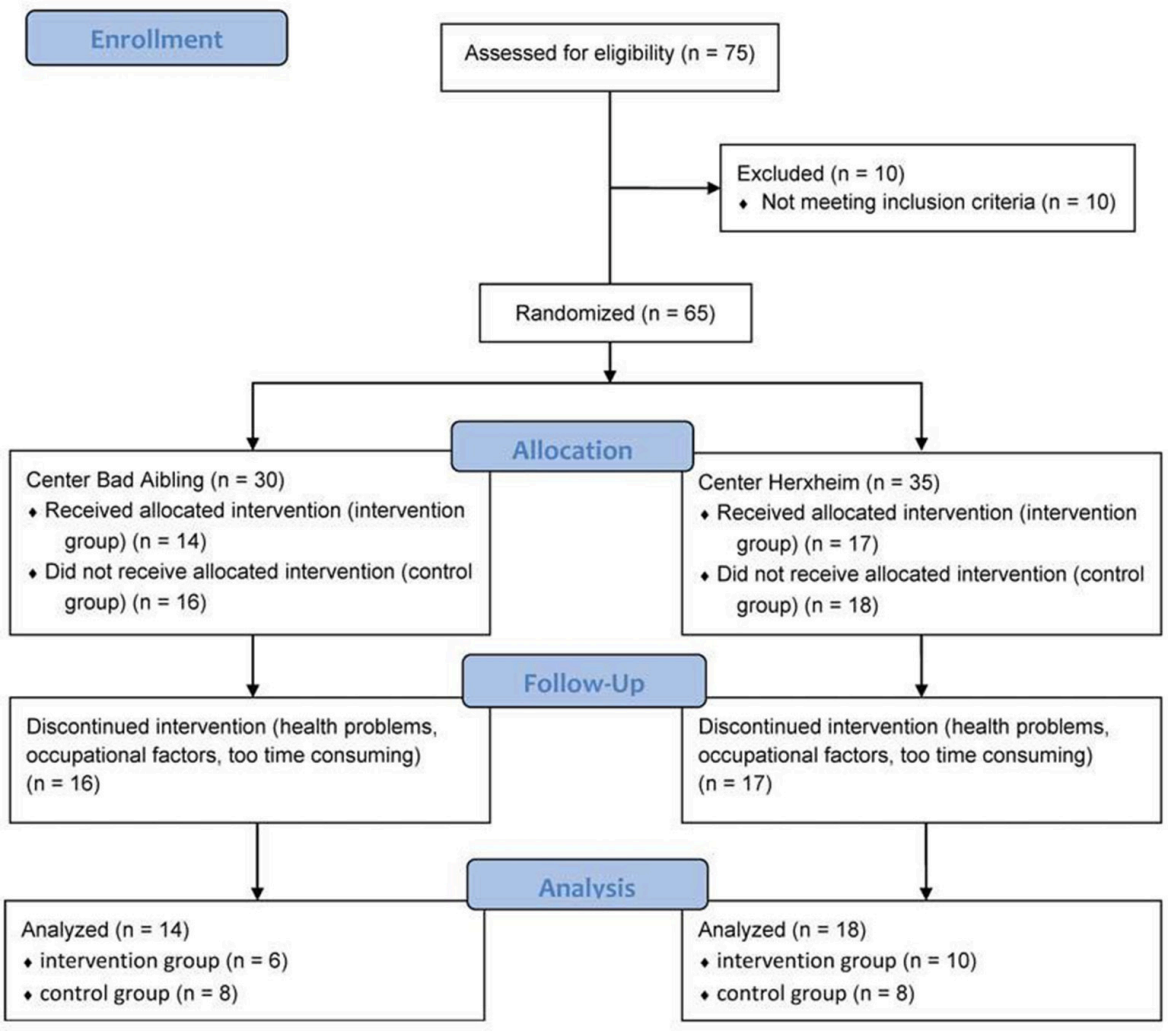
Flow chart of participants throughout course of the study. Retrieved from Gatterer et al. (10); (http://www.karger.com/?doi=10.1159/000431157).

Participants were recruited at two study sites (Bad Aibling and Herxheim, Germany) by way of notices at the two rehabilitation centers as well as by calls in local media. 30 subjects in Bad Aibling (21 females, 9 males) and 35 subjects in Herxheim (19 females, 16 males) signed up for the study and were eligible for inclusion, e.g., BMI > 30 kg/m^2^ and the ability to perform moderate intensity physical activity (adequate exercise workload and no appearance of orthopedic problems). Exclusion criteria were class >3 of the New York Heart Association (NYHA), stroke or myocardial infarction within 6 months prior to study start, malignant hypertension, instable angina pectoris, chronic kidney disease, pregnancy, and a drop of oxygen saturation <70% during 90 min at a simulated altitude of 4,500 m (pre-tested in a normobaric hypoxic chamber). All subjects provided written informed consent of procedures and study goals before performing measurements. Participants were randomly assigned to either a control group (CG, *n* = 34) or an intervention group (IG, *n* = 31). A flow chart of subject participation throughout the study is displayed in Figure 1.

The study was performed in conformity with the ethical standards laid down in the declaration of Helsinki. The ethical committees of the province of Salzburg and University of Salzburg approved the study protocol. It is registered as clinical trial at the WHO and the German Clinical Trial Register under number DRKS00005479.

### Procedures

A randomized, single-blinded, placebo-controlled intervention study was performed simultaneously at two study sites. One center was located in Bad Aibling at approximately 490m above sea level and the other center in Herxheim at approximately 130 m above sea level. Both centers provided experienced staff in exercise training at hypoxia and sham hypoxia as well as one skilled physician and assistant. The study protocol included a baseline examination, an 8-month intervention with an intermediate examination after 3 months and a final examination after 8 months. The CG exercised and rested in normoxia whereas the IG exercised and rested in normobaric hypoxia. Normobaric hypoxia was provided by an oxygen expulsion system (Low Oxygen Systems; Berlin-Buch, Germany). The expulsion system (Oxygen exchange through nitrogen) allows a mixture of fresh air to keep controlled CO_2_ levels comparable to the levels measured in hypobaric hypoxia. Blinding of participants was done by covering any display in the hypoxic rooms and running the ventilation system at the same power with closed windows in both study groups. The success of blinding was assessed by interviewing the subjects during intervention.

### Laboratory assessment of mRNA expression

Before the start of the study, after 3 months and at the end of the intervention after 8 months, laboratory assessment was performed. Abdominal subcutaneous fat biopsies were taken from subjects at the three appointed time points on a day without training session. Tissue samples were immediately frozen in liquid nitrogen and then stored at −80°C until RNA extraction. The staff of an accredited laboratory of the Medical University of Innsbruck was blinded to conditions and conducted analysis by using the laboratory technique of quantitative real-time polymerase chain reaction (qPCR) in order to determine RNA-ratios of leptin, visfatin and adiponectin. RNA was isolated by using RNeasy Lipid Tissue Mini Kit (Qiagen, Hilden, Germany, cat. no. 74804), following the company's instruction manual. The quality of isolated RNA was assessed via spectrophotometry based on OD-ratio (optical density) 260/280 (Beckman DU 640 Spectrophotometer). Samples were normalized using the reference gene glyceraldehyde-3-phosphate dehydrogenase (GAPDH). cDNA synthesis was performed according to manufacturer's handbook (Qiagen Omniscript RT Kit, cat. no. 205113). cDNA levels of respective target genes were determined by SYBR Green-based real-time PCR using Luna Universal qPCR Master Mix (New England Biolabs, Frankfurt, Germany, cat. no. M3003L). A TaqMan probe-based real-time PCR was used for GADPH (Luna Universal Probe qPCR Master Mix, New England Biolabs, cat. no. M3004S), subsequently, the manufacturers recommended PCR conditions were applied. For primer sequences of target genes, pre-designed primer-assays of Bio-Rad (Bio-Rad, Vienna, Austria) were used: qHsaCED0047448 (ADIPOQ), qHsaCID0017538 (LEP), qHsaCED0043104 (NAMPT). The primer sequence of GAPDH was TaqMan Assay Human GADPH (Applied Biosystems/Thermofisher, cat. no. 4310884E). Specificity of PCR products was confirmed by melt curve analysis. Results are presented as target gene-cDNA/GAPDH cDNA ratio.

### Additional measurements

Additional methodical information is also displayed in our previous study from this trial (10). Before the start of the study, after 5 weeks, after 3 months and at the end of the intervention, anthropometry and body composition were assessed. Additional laboratory assessment and performance testing was conducted before the start of the study, after 3 months and at the end of the intervention. Anthropometric data included weight and height measures to the nearest 0.1 kg and 0.5 cm and hip and waist circumference measures to the nearest 0.5 cm. Body composition was determined via bioelectrical impedance analysis (OMRON BF511 T Monitor, Healthcare Co., Kyoto, Japan) for estimation of total body fat and muscle mass. The standard error of the estimate (SEE) of the device is 3.5% (instruction manual, OMRON BF511, technical data). Measurements were completed according to the manufactures' guidelines. Additional laboratory assessment from venous blood samples were performed in accredited laboratories and included the determination of glucose concentration (G), glycated hemoglobin (HbA1c), high-density lipoprotein (HDL), total cholesterol (CHOL) and triglycerides (TG). Prior to performance testing, resting ECG records and blood pressure measurements were taken. Performance testing consisted of an incremental exercise test to exhaustion. The test was carried out on a cycle ergometer (Ergo-Fit Cycle 3000 MED, Ergo Fit, Pirmasens, Germany) and implemented cycling for 2 min at 5 Watt (W). Then work rate was increased by 5W every 12 s. Heart rate and gas exchange variables including ventilation (VE), oxygen uptake (VO_2_) and carbon dioxide production (VCO_2_) were recorded using an ergospirometric device (MetaVital, Cortex Biophysik Gmbh, Leipzig, Germany). The incremental exercise test was equally repeated after 3 months (10).

### Intervention

Fifty two training sessions during the 8-months study period had to be completed. Subjects exercised twice a week for 90 min with a following 90 min resting period in a normobaric hypoxic room. The IG exercised at a FiO_2_ of 14.0 ± 0.2%, which corresponds to an altitude of about 3,500 m and rested at a FiO_2_ of 12.2 ± 0.3%, equivalent to approximately 4,500 m. The CG was constrained to the same protocol in the same chambers under normoxic conditions. All participants exercised at a moderate intensity of 65–70% of the maximal heart rate corresponding to the intensity that evokes maximal fat oxidation rates in the untrained (i.e., 47–52% of VO_2max_) (25). For exercising, participants could choose between a cycle ergometer, a treadmill or a cross trainer. When treadmill or cross trainer were used, the target heart rate was increased by 10 beats/min (26). After 3 months, target heart rate was adapted according to the outcome of the second incremental exercise test. During the study period, participants were counseled to use the training regime and not to change their usual physical activity or nutritional habits.

### Statistical analysis

Based on our study from Netzer et al., a power analysis proclaimed that a group of 16 persons each would be enough statistical power to show significance in differences of body weight and laboratory parameters between IG and CG (6). In order to evaluate mean differences among groups, a one-way analysis of variance (ANOVA) was performed. *Post-hoc t*-tests were used for individual group comparison. All results are presented as mean ± standard deviation. Statistical significance was set as *p* < 0.05 for all analyses.

## Results

Thirty two subjects (16 persons in IG as well as in CG) were able to finish all 52 training sessions during the 8-months trial. Thirty three participants (18 females and 15 males) dropped out during the intervention period due to occupational factors, time-dependent problems or health issues (not related to the study protocol) (10). Overall, there were 22 female participants and 10 males (IG: 12 females, 4 males; CG: 10 females, 6 males). 60% of subjects guessed their group incorrectly, showing that blinding was successful. In both groups, no severe adverse health effects were reported.

In the hypoxia group (IG), the average age of participants was 50.3 ± 10.3 years with an average BMI of 37.9 ± 8.1 kg/m2. The normoxia group (CG) had an average age of 52.4 ± 7.9 years with an average BMI of 36.3 ± 4.0 kg/m2. Participants exercised at a mean intensity corresponding to 68.3 ± 6.3% of the HR_peak_. There was no difference in heart rate and gas exchange variables between the two incremental exercise tests regarding time, group or gender effect. An improvement over time could be detected in the reduction of body weight (*p* = 0.005), BMI (*p* = 0.018) waist and hip circumference (*p* = 0.001 and *p* = 0.016), as well as peak power output (P_peak_) (*p* = 0.002) with no differences between groups and no gender effect (10). *Post-hoc* tests of Gatterer et al., showed, that body weight decreased until month 3 and then stabilized. Systolic blood pressure improved over the 8-months intervention (*p* = 0.033) with no group differences and no gender effect. The improvements in P_peak_ did not correlate with metabolic risk factors. Changes in HDL correlated with reduced fat mass and increased muscle mass (*r* = −0.427 and *r* = 0.421, respectively; *p* < 0.05), but did not show differences regarding time or groups. Changes in triglycerides tended to correlate with changes of waist circumference and waist-to-hip ratio (*r* = 0.378 and *r* = 0.346, respectively; 0.05 < *p* < 0.1) but did not show differences regarding time or group (10).

**Figure 2:**
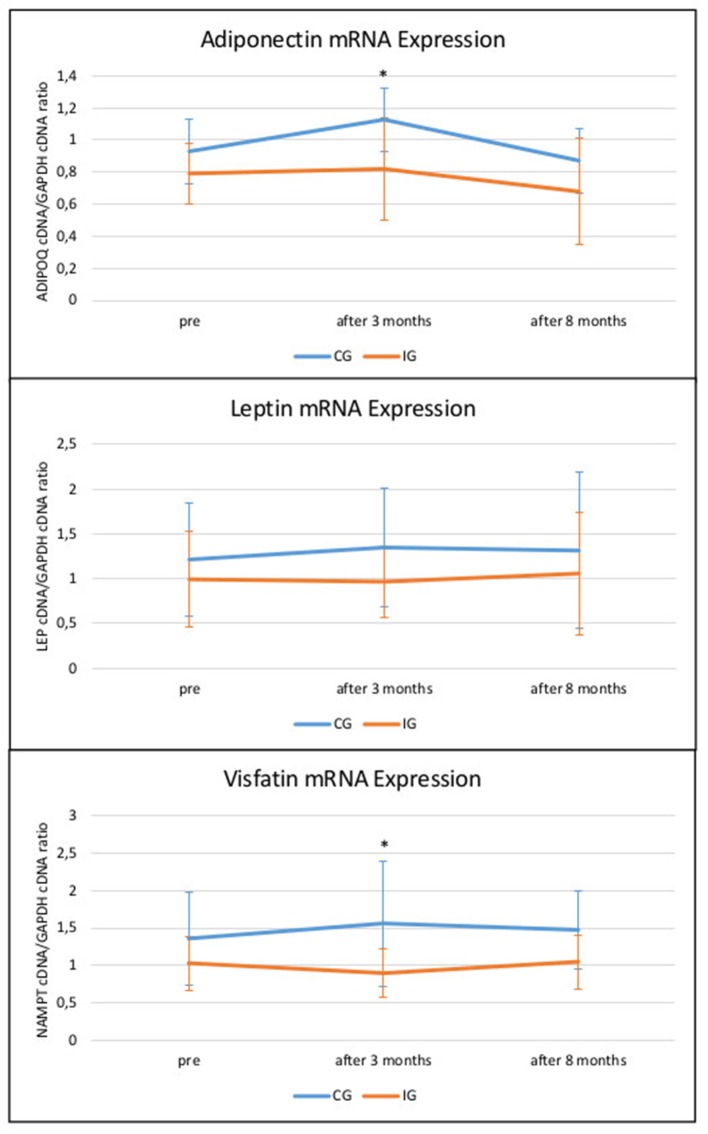
Changes of target gene's mRNA expression (±SD) over the 8-months intervention period. x-axis shows time points of subcutaneous adipocyte extraction before (pre), after 3 months and after 8 months of intervention; y-axis values display target gene's cDNA/reference gene GAPDH cDNA ratios; *p-value < 0,05; CG, control group; IG, intervention group.

Statistical analysis showed no differences in leptin mRNA expression via ANOVA (Table 1). There was no significant difference regarding time in adiponectin mRNA expression, but a center difference was observed between groups (*p* = 0.029). Baseline investigation already showed lower levels in IG, which continued to be lower in IG than in CG throughout measured time points with lowest levels measured after 8 months in IG (Figure 2).

**Table 1:** Levels of target gene's mRNA expression over the 8-months intervention period.

	**IG**				**CG**				**All**				**ANOVA**, ***p*****-value**		
	***n***	**Pre**	**3 months**	**Post**	***n***	**Pre**	**3 months**	**Post**	***n***	**Pre**	**3 months**	**Post**	**Main effect time**	**Main effect group**	**Interaction time × group**
Adiponectin	16	0.79 ± 0.19	0.82 ± 0.32	0.68 ± 0.33	16	0.93 ± 0.46	1.13 ± 0.53	0.87 ± 0.48	32	0.86 ± 0.35	0.97 ± 0.46	0.77 ± 0.41	0.169	0.029*	0.702
Leptin	16	0.99 ± 0.53	0.96 ± 0.39	1.05 ± 0.68	16	1.21 ± 0.63	1.35 ± 0.66	1.31 ± 0.87	32	1.10 ± 0.59	1.15 ± 0.57	1.18 ± 0.78	0.878	0.111	0.69
Visfatin	16	1.02 ± 0.36	0.89 ± 0.32	1.05 ± 0.36	16	1.36 ± 0.62	1.56 ± 0.84	1.47 ± 0.53	32	1.12 ± 0.53	1.23 ± 0.71	1.25 ± 0.49	0.075	0.014*	0.052

Time points of subcutaneous adipocyte extraction before (pre), after 3 months and after 8 months (post) of intervention; Adiponectin cDNA/GAPDH cDNA ratio ± standard deviation (SD), Leptin cDNA/GAPDH cDNA ratio (± SD), Visfatin cDNA/GAPDH ratio (± SD);

* *p-value < 0.05; IG, intervention group; CG, control group; n, group size*.

Likewise, there was a significant difference found in visfatin mRNA expression between groups (*p* = 0.014) as well as a trend of the effect time (*p* = 0.075) toward lower levels of visfatin in IG after 3 months and a slight elevation in IG after 8 months (Figure 2). There was no sex difference regarding the effect of the intervention.

## Discussion

To the extent of our knowledge, we conducted the first long-term and two-center randomized sham controlled training intervention study with obese subjects in simulated hypoxia. Our main findings confirm the hypothesis that long-term moderate intensity exercise under hypoxic exposure does not change levels of leptin. However, adiponectin and visfatin reached elevated levels in the normoxia group after 3 months but did not substantially change after 8 months compared to baseline measurement.

As previously reported by us, the same cohort showed that moderate exercise in hypoxia does not enhance loss of body weight or improve metabolic markers more than with resting and exercising in normoxia alone (10). In accordance with results of Engfred et al., adaptations of lipid metabolism to a similar stimulus (i.e., hypoxia dose, exercise intensity and duration) might be diminished after 3 months and lead to a stabilization of effects (27). A different explanation for the effect could be an upper limit for the degree of endocrine factors and their adaption to exercise. Hypoxic as well as normoxic exercise could provoke comparable adaptions of the hormones reflecting fat metabolism (27). Leptin as an essential regulator for lipid metabolism has been mostly investigated in normal-weight subjects at short-term altitude exposure and its circulating levels merely showed increased or unchanged results with (28, 29) and without (19, 30) significant weight loss depending on caloric intake, hypoxic dose and exercise intensity. In obese persons, literature on circulating leptin levels in hypoxia combined with exercise as well as data on leptin gene expression is scarce. Lippl et al. detected elevated circulating leptin levels under short-term hypoxia and suggested that leptin seems to be stimulated by hypoxia in high altitude despite the reduction of body weight (8). This might be because of hypoxia's induction of leptin gene expression via HIF-1α (31). When measured in extracted and cultivated human adipocytes under moderate hypoxia, elevated levels of leptin gene expression lasted for more than 14 days with a peak level at day 10 (7). In the long term, our data shows unaltered leptin mRNA expression during exercise at simulated altitude for an extended period of 8 months. Presumptively, hypoxia and exercise have a synergistic effect in stimulating leptin production in adipose tissue only for a short time period.

The effect of short term exercise on increased production of adiponectin in adipose tissue has been previously shown (32, 33). This effect appears to be overridden by long-term hypoxic exposure. Our results showed significantly lower levels of adiponectin mRNA expression in obese subjects training in hypoxia than those exercising in normoxia. However, in our study, levels of adiponectin mRNA expression decreased after the 8 months' intervention period in both groups, which suggests an adaption of mechanisms. The enhancing effect of HIF-1α on adiponectin does not seem to be present when exercising and resting in hypoxia twice a week over the duration of 8 months. Obese subjects exercising in hypoxia under caloric restriction might have shown a different outcome in terms of adiponectin, as Barnholt et al. reports no drop of adiponectin at altitude when testing healthy subjects for 3 weeks in hypoxia (4,300 m) (19). Studies with animal models further suggest an adiponectin-owed improvement of oxygen delivery during times of hypoxic stress (34). Exposure of adipose tissue to extremely high or low oxygen partial pressure (pO_2_) leads to oxidative stress, which can cause further inflammation. Due to the correlation of adiponectin and microvascular flow, it could be possible, that the role of adiponectin regarding energy homeostasis is secondary to its' regulation of the microvasculature at high altitude (34).

Adiponectin and visfatin both enhance modulation of inflammatory processes as adiponkines and share common properties. For that, visfatin was expected to show similar results as adiponectin in obese subjects. Nevertheless, in animal studies, visfatin mRNA expression was found to be up-regulated when rat hepatic stellate cells were exposed to hypoxia (23). The accumulation of visfatin also seems to correlate with the expression of HIF-1α, which would be an approach to explain the significant difference of interaction between time and group in the present study. Levels of visfatin mRNA expression were lower in the intervention group and tended to increase over the duration of 8 months, possibly because of endocrine adjustment to the unchanged hypoxic stimulus. Further studies will be necessary to understand visfatin's function in obese subjects exercising in hypoxia during a long-term intervention.

Testing adipokines on mRNA level only might seem as limitation of the current study. However, after detecting no significant differences regarding HDL or triglycerides in blood circulation, we assumed that further measurements on protein level would have not strengthened our study.

Furthermore, it is to notice that levels of target genes' mRNA expression were already elevated at baseline in the control group. For this, we have no explanation.

We regard the lack of report concerning food intake and additional recreational exercise of subjects as a further limitation of this study, as these factors could potentially affect endocrine changes. Even though participants were instructed not to change their usual eating habits and daily physical activity, some alteration might have occurred outside of study protocol. The dropout rate of our study population added up to 51.7%, which matches other long-term exercise intervention trials (35) and studies requiring a change of lifestyle (36–38).

## Conclusion

Several weeks' moderate exercise of obese subjects in moderate hypoxia leads to significant more weight loss compared to the same training in normoxia, but this effect does not continue and hold up for several months. Whereas increased leptin in the first 2 weeks is probably the cause for this additional hypoxia effect, leptin mRNA-levels are not elevated compared to normoxia after several months. In opposition, for positive health effects recognized adipokines, namely adiponectin and visfatin, are elevated in mRNA-expression after three months training in obese persons in normoxia but not in hypoxia. We suspect, that a several days or few weeks moderate hypoxic exercise program might have positive effects for obese persons, whereas a several months hypoxic exercise program does not seem to be superior in its health preventive effects over exercise in normoxic conditions. Nevertheless, the additional effect of hypoxia generates a higher cardiac output despite less effort (i.e., performance on an ergometer) compared to normoxia. This effect is especially beneficial for obese people, due to enhanced cardiovascular circulation and reduced strain on joints. A higher resting heart rate from the start causes enhanced cardiovascular circulation, which creates faster oxygen supply in untrained musculature. Therefore, we suggest starting with moderate exercise training in hypoxia to ease the beginning and continue exercising after several weeks in normoxia in order to be able to obtain exercising on a regular basis and acquire a long-term change of lifestyle.

## Author contributions

LR: data analysis, writing manuscript; MH: laboratory assessment and evaluation, writing manuscript; SP: data analysis, writing manuscript; SK: laboratory assessment and evaluation; CE: collecting laboratory data; SH: acquiring data; HG: data analysis; NN: designing research strategy, acquiring data, writing manuscript.

### Conflict of interest statement

The authors declare that the research was conducted in the absence of any commercial or financial relationships that could be construed as a potential conflict of interest.
